# An LPS-Immobilized Affinity Screening Platform Identifies Lipopolysaccharide Binding Phytochemicals from *Taraxacum mongolicum*

**DOI:** 10.3390/biology15151264

**Published:** 2026-08-01

**Authors:** Dandan Zhang, Ruinan Li, Ruoliang Wang, Yuhang Tang, Langjie Luosang, Xiaoning Zhao, Qian Li, Xinfeng Zhao, Yinku Liang

**Affiliations:** 1 Provincial-Ministry Joint State Key Laboratory of Qinba Biological Resources and Ecological Environment, Shaanxi Provincial Key Laboratory of Resource Biology, School of Biological Sciences and Engineering, Shaanxi University of Technology, Hanzhong 723000, China; zhangdandan@snut.edu.cn (D.Z.); 18195412164@163.com (R.L.); wangruoliang2024@163.com (R.W.); qijixiaoyu2025@163.com (Y.T.); 18308007255@163.com (L.L.); jasminezxnsx@msn.com (X.Z.); 2 The College of Life Sciences, Northwest University, Xi’an 710069, China; 20185365@nwu.edu.cn (Q.L.); zhaoxf@nwu.edu.cn (X.Z.)

**Keywords:** *Taraxacum mongolicum*, lipopolysaccharide, Ag@SiO_2_, affinity screening, UPLC-Q-Orbitrap HRMS/MS, SPR, antibacterial activity

## Abstract

Lipopolysaccharide (LPS) is a major component of the outer membrane of Gram-negative bacteria and a key factor involved in the induction of host inflammatory responses. In this study, an LPS-immobilized Ag@SiO_2_ affinity screening platform was established to enrich potential LPS-binding constituents from an extract of *Taraxacum mongolicum*, followed by identification and validation using high-resolution mass spectrometry, surface plasmon resonance (SPR), and antibacterial assays. Affinity enrichment coupled with mass spectrometric analysis resulted in the annotation of 14 candidate constituents, primarily phenolic acids and flavonoids. All six representative compounds exhibited detectable interactions with immobilized LPS. Among them, chlorogenic acid and cichoric acid showed relatively strong apparent affinities, with $K_{D}$ values of 7.42 × 10^−9^ and 2.63 × 10^−8^ M, respectively. Antibacterial assays indicated that these compounds generally exhibited limited direct growth-inhibitory effects against *Escherichia coli* and *Shigella* spp., with cichoric acid showing an MIC of 1024 μg/mL against both tested bacteria. These findings identify chlorogenic acid and cichoric acid as notable candidate LPS-binding constituents of *T. mongolicum*. This study provides a methodological basis for screening LPS-binding natural small molecules from complex plant extracts.

## Simple Summary

Lipopolysaccharide (LPS), a component of the outer membrane of Gram-negative bacteria, is a major trigger of severe inflammatory responses. In this work, we used an LPS-immobilized nano-affinity platform to screen and identify 14 potential LPS-binding natural compounds from dandelion (*Taraxacum mongolicum*), which were mainly phenolic acids and flavonoids. Surface plasmon resonance analysis verified that chlorogenic acid and cichoric acid exhibited relatively strong binding to LPS. Antibacterial tests further revealed that phenolic acids generally showed stronger inhibitory effects against bacteria than flavonoids, although all tested compounds had only limited direct activity against *Escherichia coli* and *Shigella* spp. This study offers a feasible approach for the targeted discovery of LPS-binding bioactive molecules from complex botanical extracts, which may support the development of new anti-infective lead compounds.

## 1. Introduction

Gram-negative bacterial infections are widely involved in various infectious diseases and represent an important etiological basis for severe inflammatory responses and sepsis [1,2]. Lipopolysaccharide (LPS), a major component of the outer membrane of Gram-negative bacteria, is not only essential for maintaining bacterial outer membrane integrity but also serves as a key pathogenic factor that triggers host inflammatory cascades and endotoxin-associated injury. LPS can induce the massive release of pro-inflammatory cytokines, thereby amplifying inflammatory responses and leading to endotoxemia, tissue injury, and even multiple organ dysfunction. Therefore, interventions against Gram-negative bacterial infections should not be limited to bacterial growth inhibition or bactericidal activity, but should also consider LPS-mediated inflammatory processes. Because targeting LPS may interfere with bacterial outer membrane homeostasis and provide a potential entry point for modulating LPS-associated inflammatory responses, LPS has become an important target for the development of anti-Gram-negative bacterial agents and LPS-targeted regulatory candidates [2,3,4,5].

*Taraxacum mongolicum* is a commonly used traditional medicinal plant that contains multiple classes of bioactive constituents, including phenolic acids, flavonoids, and coumarins, and exhibits diverse pharmacological activities such as anti-inflammatory, antibacterial, and antioxidant effects [6,7,8]. However, current studies on the active constituents of *T. mongolicum* have mainly focused on crude extracts or specific fractions. Whether *T. mongolicum* contains small-molecule constituents capable of directly recognizing LPS, and how such binding behavior is associated with its antibacterial or inflammation-modulating potential, remains insufficiently investigated. Conventional bioactivity-guided isolation strategies are often labor-intensive, time-consuming, and inefficient, making it particularly difficult to accurately identify LPS-targeted active molecules from complex natural product systems [9,10]. In addition, theoretical predictions based solely on molecular docking lack direct biophysical evidence and cannot fully reveal the authentic interaction characteristics between natural products and LPS. Therefore, establishing an integrated research strategy that combines efficient screening, binding validation, and functional evaluation is of great significance for clarifying the material basis of plant-derived LPS-targeted active constituents.

This study aimed to identify and validate potential LPS-binding phytochemicals from *Taraxacum mongolicum* using an LPS-immobilized Ag@SiO_2_ affinity screening platform. Candidate constituents were affinity-enriched from the plant extract and chemically characterized, followed by evaluation of their interactions with immobilized LPS and preliminary antibacterial activity. Target-directed biochromatographic platforms have similarly been applied to screen receptor-selective constituents from complex medicinal-plant matrices, followed by SPR-based binding evaluation and functional validation [11]. Bio-affinity ultrafiltration coupled with LC–MS has previously been applied to identify target-binding constituents from complex medicinal-plant matrices, including mulberry leaves and *Azadirachta indica* extracts [12,13]. In contrast, the present study employed an LPS-immobilized Ag@SiO_2_ platform for affinity enrichment and further integrated SPR-based binding validation. Moreover, whereas previous SERS studies have mainly used plasmonic substrates for the detection of LPS or bacterial endotoxins [14,15], the present work extended this application to the characterization of LPS immobilization and the affinity enrichment of candidate constituents from a complex natural-product matrix. Collectively, this study establishes an integrated workflow for discovering potential LPS-binding natural small molecules from complex plant extracts.

## 2. Materials and Methods

### 2.1. Materials, Reagents, and Herbal Samples

The whole plants with roots of *Taraxacum mongolicum* were purchased from Guangzhou Juntang Technology Co., Ltd., Guangzhou, China. The plant material was botanically authenticated by Professor Yinku Liang of Shaanxi University of Technology. Lipopolysaccharide (LPS, purity 98%) was obtained from Shanghai Yueteng Biotechnology Co., Ltd., Shanghai, China. Silver nitrate (AgNO_3_), sodium citrate, polyvinylpyrrolidone (PVP), methanol, formic acid, ammonia solution, MES buffer, and other reagents were of analytical or chromatographic grade. Anhydrous ethanol and deionized water were used for nanomaterial preparation and sample processing. Reference standards, including caffeic acid, chlorogenic acid, monocaffeoyltartaric acid, luteolin, quercetin, rutin, ferulic acid, *p*-coumaric acid, cichoric acid, luteolin-7-*O*-glucoside, hyperoside, isoquercitrin, and esculetin, with purities greater than 99.8%, were purchased from Chengdu Preferred Biotechnology Co., Ltd., Chengdu, China.

### 2.2. Instruments and Analytical Equipment

The main instruments used in this study included an ultra-performance liquid chromatography–Q-Orbitrap high-resolution mass spectrometry system (UPLC-Q-Orbitrap HRMS, Thermo Fisher Scientific, Waltham, MA, USA), a surface plasmon resonance instrument (Biacore X100, Cytiva, Uppsala, Sweden), a transmission electron microscope (TALOS F200X G2, Thermo Fisher Scientific, Waltham, MA, USA), a magnetic stirrer (78-1, Jiangyin Baoli Scientific Research Instrument Co., Ltd., Jiangyin, China), a rotary evaporator (R-3001, Zhengzhou Great Wall Scientific Industrial and Trade Co., Ltd., Zhengzhou, China), a freeze dryer (LGJ-10, Beijing Sihuan Scientific Instrument Factory Co., Ltd., Beijing, China), a benchtop centrifuge (TGL-20M, Shanghai Feiqiaer Analytical Instrument Co., Ltd., Shanghai, China), an electronic analytical balance (XPR106DU, Mettler Toledo, Greifensee, Switzerland), and an ultrasonic cleaner (YL0203-28, Dongguan Kangshijie Ultrasonic Technology Co., Ltd., Dongguan, China).

### 2.3. Preparation of Ag Nanoparticles

Ag nanoparticles (Ag NPs) were prepared using a chemical reduction method. Briefly, 0.36 g of polyvinylpyrrolidone (PVP), 0.20 g of sodium citrate, and 0.30 g of silver nitrate (AgNO_3_) were separately dissolved in 10 mL of deionized water, with the AgNO_3_ solution freshly prepared before use. Subsequently, 370 mL of deionized water was transferred into a conical flask and heated under magnetic stirring. After the system was uniformly stirred, the AgNO_3_ solution was first added and allowed to react for 5 min. The PVP solution was then introduced, and the mixture was further heated to 80 °C. Once the temperature reached 80 °C, the sodium citrate solution was added, and the reaction was maintained for 1 h. After completion, the reaction mixture was cooled to room temperature, and the resulting suspension was centrifuged at 8000 rpm and 4 °C for 10 min. The supernatant was discarded, and the precipitate was washed twice with distilled water and twice with anhydrous ethanol. The collected Ag NPs were stored at 4 °C until further use.

### 2.4. Fabrication of Ag@SiO_2_ Core–Shell Nanostructures

Ag@SiO_2_ core–shell nanostructures were further fabricated based on the prepared Ag NPs. The obtained Ag NPs were centrifuged and washed once, followed by washing twice with anhydrous ethanol. The precipitate was collected, dispersed in 50 mL of anhydrous ethanol, and ultrasonicated for 10 min to obtain a homogeneous dispersion. Subsequently, 5 mL of ammonia solution was added, and tetraethyl orthosilicate (TEOS) was slowly added dropwise under continuous stirring. The reaction was carried out at room temperature for 4 h. After completion, the product was collected by centrifugation and washed with anhydrous ethanol to remove unreacted reagents and by-products, yielding Ag@SiO_2_ core–shell nanostructures. The resulting material was used for subsequent surface amino functionalization and LPS immobilization experiments.

### 2.5. Amino-Functionalization of Ag@SiO_2_ Nanostructures

The surface of the Ag@SiO_2_ core–shell nanostructures was amino-functionalized using 3-aminopropyltriethoxysilane (APTES). Briefly, APTES was mixed with anhydrous ethanol at a volume ratio of 1:100 to prepare a 1% APTES solution. The Ag@SiO_2_ nanoparticles were dispersed in anhydrous ethanol at a concentration of approximately 0.5 mg/mL and ultrasonicated for 10 min to improve particle dispersion and remove surface impurities. The APTES solution was then added to the dispersion, and the mixture was stirred at room temperature for 2 h. During the reaction, nitrogen was introduced to reduce the hydrolysis and self-polymerization of APTES in air. After the reaction, the mixture was centrifuged at 8000 rpm for 10 min, and the supernatant was discarded. The precipitate was washed three times with anhydrous ethanol and once with deionized water to remove unbound APTES. Finally, the product was dispersed in an appropriate buffer and stored for subsequent use.

### 2.6. Immobilization of Lipopolysaccharide on the Plasmonic Core–Shell Surface

LPS was immobilized onto the surface of amino-functionalized Ag@SiO_2_ core–shell nanostructures using EDC/NHS-mediated coupling. Briefly, LPS was dissolved in MES buffer at pH 6.0 and fully dispersed. EDC and NHS were then added to the LPS solution at a molar ratio of 1:1 to activate the reactive carboxyl groups in LPS, and the reaction was allowed to proceed at room temperature for 30 min. After activation, amino-functionalized Ag@SiO_2_ was added to the reaction system, and the pH was adjusted to 7.0–8.0. The mixture was further reacted at room temperature for 4 h to achieve covalent conjugation between LPS and the amino-functionalized core–shell surface. After the reaction, glycine was added to terminate the coupling reaction and quench residual active intermediates. Subsequently, unbound small-molecule by-products were removed by centrifugation, and the resulting precipitate was washed to obtain the LPS-immobilized Ag@SiO_2_ core–shell nanoscreening platform.

### 2.7. Characterization of the LPS-Immobilized Core–Shell Platform

Ag NPs, Ag@SiO_2_, amino-functionalized Ag@SiO_2_, and LPS-immobilized Ag@SiO_2_ were characterized by transmission electron microscopy (TEM) and energy-dispersive X-ray spectroscopy (EDS) to evaluate the morphological evolution and elemental composition changes during the stepwise construction of the LPS-immobilized core–shell platform. Prior to TEM analysis, dispersions of the nanomaterials obtained at each preparation stage were diluted with water, dropped onto carbon-coated copper grids, and naturally dried at room temperature. Particle morphology, dispersion state, core–shell structure, and particle size changes were analyzed based on TEM images. The particle size distribution of each sample was statistically evaluated using ImageJ (v1.54f) software by randomly selecting particles from each group. EDS analysis was performed to determine the elemental composition of Ag, Si, O, C, and N in samples collected at different modification stages.

### 2.8. Preparation of Taraxacum mongolicum Extracts

The crude extract of *T. mongolicum* was prepared using an ultrasound-assisted extraction method. Briefly, the dried herbal material of *T. mongolicum* was pulverized into powder and extracted with 70% ethanol at a solid-to-liquid ratio of 1:10 (*w*/*v*) under ultrasonication at room temperature for 60 min. After extraction, the solution was vacuum-filtered, and the filtrate was concentrated using an R-3001 rotary evaporator (Zhengzhou Great Wall Scientific Industrial and Trade Co., Ltd., Zhengzhou, China) at 55 °C under reduced pressure (−0.09 MPa) to obtain a wet crude extract. The wet extract was subsequently freeze-dried, and the resulting dried extract was stored at −20 °C until further use. The extract was stored at −20 °C until further use.

### 2.9. Affinity Screening of LPS-Binding Phytochemicals from Taraxacum mongolicum

The *T. mongolicum* extract was prepared as a 1 mg/mL sample solution for LPS-targeted affinity screening. Briefly, 0.1 g of LPS-immobilized Ag@SiO_2_ core–shell nanomaterials was added to 50 mL of the *T. mongolicum* extract solution and incubated at 37 °C for 2 h to facilitate sufficient binding between potential active constituents in the extract and immobilized LPS. After incubation, the mixture was centrifuged at 8000 rpm for 10 min, and the supernatant was discarded. The resulting precipitate was washed three times with PBS buffer, with centrifugation performed after each washing step, to remove unbound constituents and nonspecifically adsorbed compounds. Subsequently, methanol was added to the washed precipitate for elution, and the mixture was shaken at room temperature for 1 h to release candidate small-molecule constituents bound to the LPS surface. After elution, the mixture was centrifuged again, and the supernatant was collected and filtered through a 0.22 μm microporous membrane for subsequent UPLC-Q-Orbitrap HRMS/MS analysis.

### 2.10. UPLC-Q-Orbitrap HRMS/MS Analysis and Identification of Screened Compounds

The eluate obtained from affinity screening was analyzed using an ultra-performance liquid chromatography–Q-Orbitrap high-resolution mass spectrometry system (UPLC-Q-Orbitrap HRMS, Thermo Fisher Scientific). Chromatographic separation was performed on a Waters Acquity UPLC HSS T3 column (2.0 mm × 100 mm, 1.7 μm). Acetonitrile was used as mobile phase A, and formic acid aqueous solution was used as mobile phase B. The gradient elution program was as follows: 0–15 min, 20% A; 15–18 min, 20% A; 18–25 min, 30% A; 25–27 min, 90% A; 27–29 min, 90% A; and 29–32 min, 5% A. The column temperature was maintained at 20 °C, the flow rate was 0.3 mL/min, and the injection volume was 5 μL.

Mass spectrometric detection was performed using an electrospray ionization source (ESI) in negative ion mode. Full-scan MS was first conducted to obtain the total ion chromatogram of the eluted components and high-resolution quasi-molecular ion information, with a scan range of *m*/*z* 50–750. Subsequently, MS/MS fragment information was acquired for candidate ions showing distinct responses in the full-scan MS spectra to assist structural identification. Candidate compounds were tentatively identified based on chromatographic retention behavior, high-resolution quasi-molecular ions, characteristic MS/MS fragment ions, and comparison with relevant literature data.

### 2.11. SPR Analysis of Compound–LPS Binding Kinetics

Surface plasmon resonance (SPR) was used to evaluate the direct binding capacity and apparent kinetic parameters of representative compounds toward immobilized LPS. The experiments were performed on a Biacore X100 system using a CM5 sensor chip (Cytiva, Uppsala, Sweden), with MES buffer as the running buffer. LPS was immobilized onto the CM5 chip surface through EDC/NHS-mediated coupling. The reference channel was subjected to the same activation and blocking procedures without LPS immobilization and was used to subtract background responses and nonspecific signals. Quercetin, cichoric acid, rutin, chlorogenic acid, luteolin-7-*O*-glucoside, luteolin, and the positive control polymyxin B were separately prepared at concentration gradients ranging from 25 to 1000 μM and injected sequentially from low to high concentrations. The flow rate was set at 30 μL/min, with an association time of 120 s and a dissociation time of 300 s. After each cycle, the chip surface was regenerated with 0.1 mM NaOH for 30 s to restore the baseline. The obtained sensorgrams were processed by reference channel subtraction, blank injection subtraction, and solvent correction. The data were then fitted using a 1:1 Langmuir binding model to obtain the association rate constant (*k*a), dissociation rate constant (*k*d), and equilibrium dissociation constant (*K*D), which were used to compare the apparent binding capacities of different compounds toward immobilized LPS.

### 2.12. SERS Analysis of Platform Construction and Affinity-Enriched Small Molecules

SERS spectroscopy was used to characterize Ag NPs, Ag@SiO_2_, Ag@SiO_2_-NH_2_, Ag@SiO_2_-NH_2_-LPS, and the LPS-immobilized nanocomposites incubated with *T. mongolicum* extract, in order to evaluate the stepwise construction of the nanoplatform, LPS immobilization, and the binding process of candidate small molecules. After sufficient dispersion, the samples were dropped onto clean glass slides and naturally dried prior to spectral acquisition. Raman/SERS measurements were performed using a 785 nm laser as the excitation source, with the laser power set at 20 mW, an objective magnification of 20×, an integration time of 3 s, and six cumulative scans for each detection point. The spectral acquisition range was 50–4000 cm^−1^. Five parallel spectra were collected for each sample to evaluate the reproducibility of the spectral signals. The acquired spectra were processed using polynomial fitting-based baseline correction to remove fluorescence background and baseline drift, followed by necessary smoothing; subsequently, the characteristic peak positions and relative intensity changes in samples at different modification stages were compared.

### 2.13. Molecular Docking Analysis

The three-dimensional structure of LPS/Lipid A was obtained from the RCSB PDB database, and the structures of candidate small-molecule compounds were obtained from the Chembook database. Both receptors and ligands were preprocessed using AutoDock Tools 1.5.7, including hydrogen addition, Gasteiger charge assignment, and conversion into PDBQT format. Before docking, the three-dimensional conformations of the small-molecule ligands were constructed and energy-minimized using Chem3D. Molecular docking was performed using AutoDock Vina 1.2.5, with the grid box set to cover the major exposed regions of LPS. Binding energy was used as the preliminary evaluation index for the docking results, with lower binding energy indicating stronger potential binding capacity between the ligand and LPS. The dominant conformation with the lowest binding energy was selected, and its binding mode was visualized using PyMOL 3.0 and Discovery Studio Visualizer 2021, with particular attention paid to intermolecular interactions, including hydrogen bonds, hydrophobic interactions, and electrostatic interactions.

### 2.14. Antibacterial Activity Evaluation

The antibacterial activities of six selected candidate compounds—chlorogenic acid, cichoric acid, rutin, quercetin, luteolin-7-*O*-glucoside, and luteolin—were evaluated against *Escherichia coli* and *Shigella* spp. The crude extract of *T. mongolicum* was not included in the antibacterial assays. After activation culture at 37 °C, the bacterial strains were inoculated into liquid medium and shaken until reaching the logarithmic growth phase, then diluted to approximately 1 × 10^6^ CFU/mL for subsequent experiments. The inhibition zone assay was used to preliminarily evaluate the inhibitory effects of each compound on bacterial growth. After the assay, the diameters of the inhibition zones were measured and compared with those of the positive and negative controls. The minimum inhibitory concentration (MIC) was determined using the broth microdilution method. The test compounds were serially diluted in 96-well plates, followed by the addition of an equal volume of bacterial suspension, and then incubated at 37 °C for 24 h. The lowest concentration showing no visible bacterial growth was defined as the MIC. Polymyxin B was used as the positive control, and the corresponding solvent was used as the negative control. All experiments were performed in triplicate. All antibacterial assays involving *E. coli* and *Shigella* spp. were conducted under standard biosafety level 2 practices in accordance with institutional biosafety regulations, with appropriate personal protective measures and aseptic techniques applied throughout the experimental procedures.

### 2.15. Statistical Analysis

Data were processed using Origin 2024 and ImageJ. Inhibition zone assays were performed in three independent experiments, and the results are presented as the mean ± standard deviation. Differences among compound groups were analyzed using one-way analysis of variance followed by Tukey’s multiple-comparison test, with *p* < 0.05 considered statistically significant. MIC values were determined using the twofold serial dilution method in three independent experiments. For SERS analysis, five replicate spectra were collected for each group to assess spectral reproducibility. SPR sensorgrams were fitted after reference-channel and blank-signal subtraction, and the resulting parameters were reported as apparent kinetic parameters.

## 3. Results

### 3.1. Construction of the LPS-Immobilized Ag@SiO_2_ Platform and TEM/EDS Characterization

**Figure 1:**
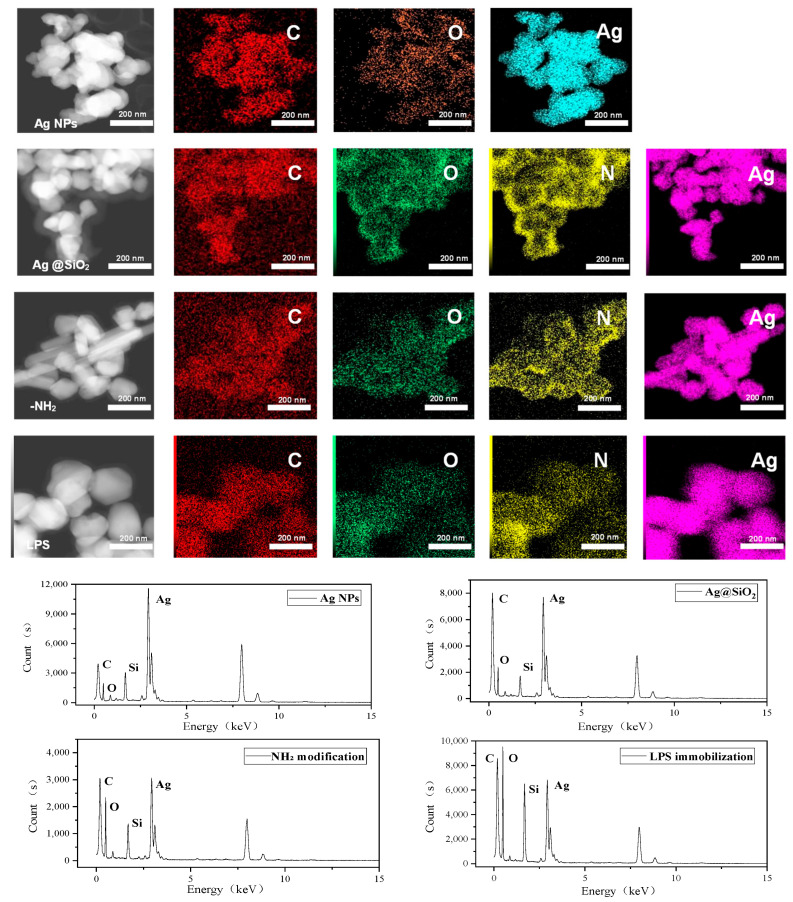
Elemental analysis of Ag@SiO_2_ nanostructures during synthesis, amino functionalization, and lipopolysaccharide conjugation.

To construct the LPS-immobilized plasmonic core–shell platform, Ag NPs, Ag@SiO_2_, Ag@SiO_2_-NH_2_, and LPS-conjugated Ag@SiO_2_ nanomaterials were sequentially prepared and characterized by TEM and EDS to evaluate their morphology and elemental composition. TEM results showed that the prepared Ag NPs exhibited a uniform quasi-spherical morphology (Figure 1), with an average particle size of 70 ± 4 nm. After SiO_2_ coating, the particles displayed a typical core–shell structure with a clear boundary between the silver core and the silica shell, and the average particle size increased to 93 ± 4 nm. After further amino functionalization, the particle size increased to 100 ± 7 nm. Following LPS conjugation, the average particle size further increased to 105 ± 6 nm. The continuous increase in particle size during the successive modification stages indicated the stepwise completion of surface functionalization, suggesting that LPS was successfully immobilized on the Ag@SiO_2_ surface.

Semi-quantitative EDS analysis indicated that the elemental composition of the samples changed progressively during platform construction. As shown in Table 1, Ag accounted for 44.15 wt% of the Ag NPs. After SiO_2_ coating, the relative Ag content decreased to 20.33 wt%, while Si and O were detected at 25.04 and 23.26 wt%, respectively, indicating the formation of a Si- and O-rich SiO_2_ shell on the particle surface. Following further amino functionalization with APTES, the N content reached 2.26 wt%, whereas the Ag, Si, and O contents were 18.36, 23.48, and 21.72 wt%, respectively, consistent with the introduction of amino-containing silane groups. After LPS immobilization, the relative contents of Ag and Si further decreased to 8.50 and 14.37 wt%, respectively, while the C, O, and N contents increased to 46.71, 26.91, and 3.51 wt%, respectively. These changes reflect an increased abundance of organic components and oxygen- and nitrogen-containing groups on the particle surface, consistent with the formation of an LPS-containing surface layer. Collectively, the semi-quantitative EDS results support the stepwise construction of the Ag@SiO_2_ platform through amino functionalization and subsequent LPS immobilization.

### 3.2. Raman Spectroscopic Verification of the Stepwise Construction of the Ag@SiO_2_ Nanoplatform and LPS Immobilization

**Figure 2:**
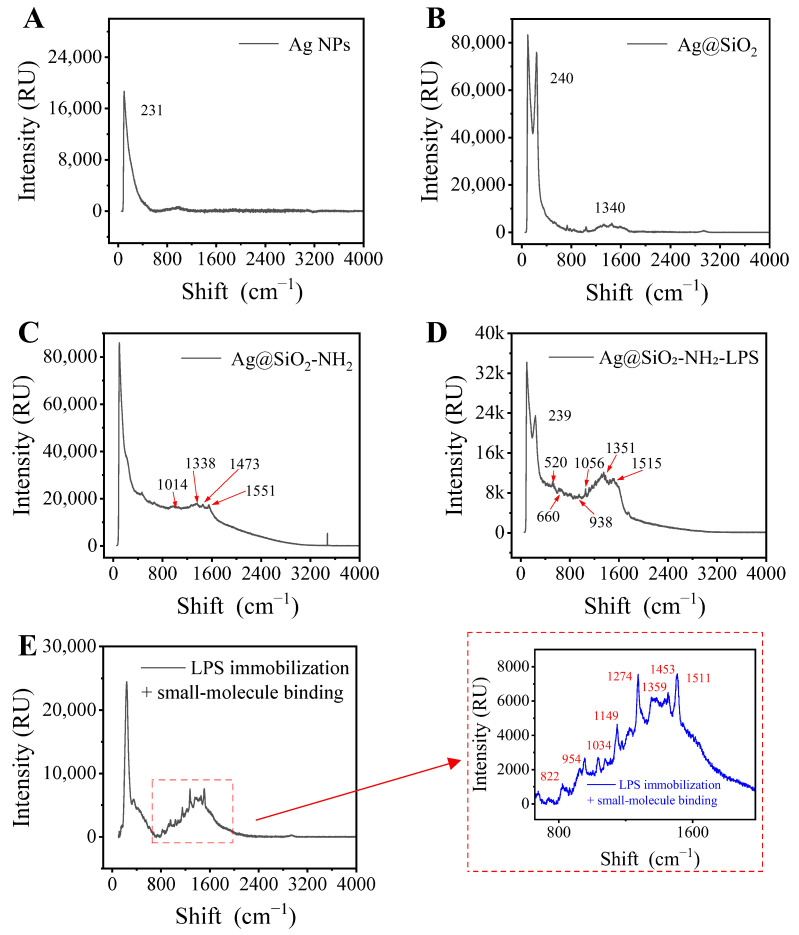
Raman spectra showing the stepwise construction of the LPS-immobilized Ag@SiO_2_ nanoplatform and subsequent small-molecule binding. (**A**) Ag NPs; (**B**) Ag@SiO_2_; (**C**) Ag@SiO_2_-NH_2_; (**D**) Ag@SiO_2_-NH_2_-LPS; (**E**) LPS-immobilized nanoplatform after small-molecule binding.

To verify the stepwise construction of the Ag@SiO_2_ nanoplatform and the LPS immobilization process, Raman spectroscopic characterization was performed on materials at different modification stages. As shown in Figure 2A, bare Ag NPs showed no obvious molecular characteristic peaks, with only background signals observed in the low-wavenumber region, indicating that they mainly served as the SERS-enhancing substrate. After SiO_2_ coating, Ag@SiO_2_ exhibited an Ag–O/Ag–O–Si-related vibrational peak at approximately 240 cm^−1^ and a weak peak near 1340 cm^−1^, suggesting the formation of the SiO_2_ shell and the Ag/SiO_2_ interfacial structure. After further amino functionalization, Ag@SiO_2_–NH_2_ showed characteristic peaks at 1014, 1338, 1473, 1551, and 3474 cm^−1^, which were associated with Si–O–Si/Si–O–C, C–N/CH_2_, CH_2_ bending, –NH_2_/–NH_3_^+^, and N–H/O–H vibrations, respectively, indicating the successful grafting of APTES and the introduction of amino groups.

After LPS immobilization, Ag@SiO_2_–NH_2_–LPS displayed new or enhanced characteristic peaks at 660, 938, 1056, 1351, and 1515 cm^−1^, which were mainly attributed to sugar-ring vibrations, C–O/C–O–C vibrations, CH_2_ vibrations, and COO^−^/N–H-related vibrations in LPS, confirming the successful immobilization of LPS onto the surface of the amino-functionalized nanoplatform. After further incubation with *T. mongolicum* extract, multiple small-molecule characteristic peaks appeared in the range of 800–1600 cm^−1^, including peaks at 822, 954, 1034, 1149, 1274, 1359, 1453, and 1511 cm^−1^. These peaks mainly corresponded to vibrations of aromatic rings, phenolic hydroxyl groups, glycosidic bonds, C–O/C–O–C bonds, and conjugated C=C skeletons, suggesting that immobilized LPS could effectively enrich potential LPS-binding constituents from *T. mongolicum* extract, including polyphenols, flavonoids, flavonoid glycosides, phenolic acids, and coumarins.

### 3.3. Targeted Affinity Screening Identifies Multiple LPS-Binding Phytochemicals from Taraxacum mongolicum

**Figure 3:**
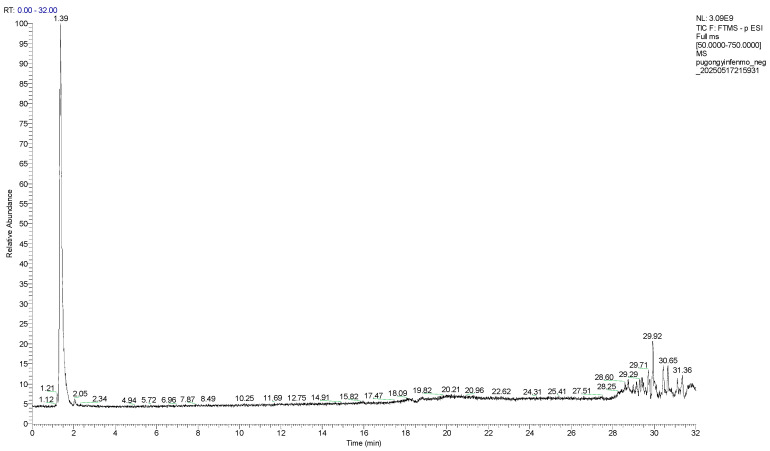
Total ion chromatogram of lipopolysaccharide-targeted active constituents identified by affinity screening.

Based on the LPS-immobilized affinity screening platform, constituents capable of binding to lipopolysaccharide in *T. mongolicum* extract were enriched, and the eluted components were analyzed by UPLC-Q-Orbitrap HRMS/MS (Figure 3). The full-scan MS results showed that, compared with the blank control, multiple candidate binding constituents with distinct chromatographic responses and mass spectrometric signals were observed in the eluate from the LPS-immobilized platform. MS/MS fragment information was further acquired for the major candidate ions, and comprehensive comparisons were performed based on chromatographic retention behavior, quasi-molecular ion peaks, characteristic fragment ions, reference standards, and relevant literature data. A total of 14 phytochemical constituents potentially capable of binding to LPS were annotated, including caffeic acid, chlorogenic acid, monocaffeoyltartaric acid, luteolin, quercetin, rutin, ferulic acid, *p*-coumaric acid, cichoric acid, luteolin-7-*O*-glucoside, hyperoside, isoquercitrin, esculetin, and behenic acid. Detailed information is provided in Table 2.

In terms of chemical structural classes, these candidate LPS-binding constituents mainly included phenolic acids and their derivatives, flavonoids, coumarins, and fatty acids. Specifically, caffeic acid, chlorogenic acid, monocaffeoyltartaric acid, ferulic acid, *p*-coumaric acid, and cichoric acid were classified as phenolic acids or phenolic acid ester derivatives; luteolin, quercetin, rutin, luteolin-7-*O*-glucoside, hyperoside, and isoquercitrin belonged to flavonoids; esculetin was classified as a coumarin; and behenic acid was categorized as a long-chain fatty acid. Overall, the candidate binding constituents were dominated by phenolic acids and flavonoids, suggesting that polyphenolic constituents enriched in *T. mongolicum* may represent an important material basis mediating its interaction with LPS.

### 3.4. Molecular Docking Suggests Potential Interaction Modes Between Representative Compounds and LPS/Lipid A

**Figure 4:**
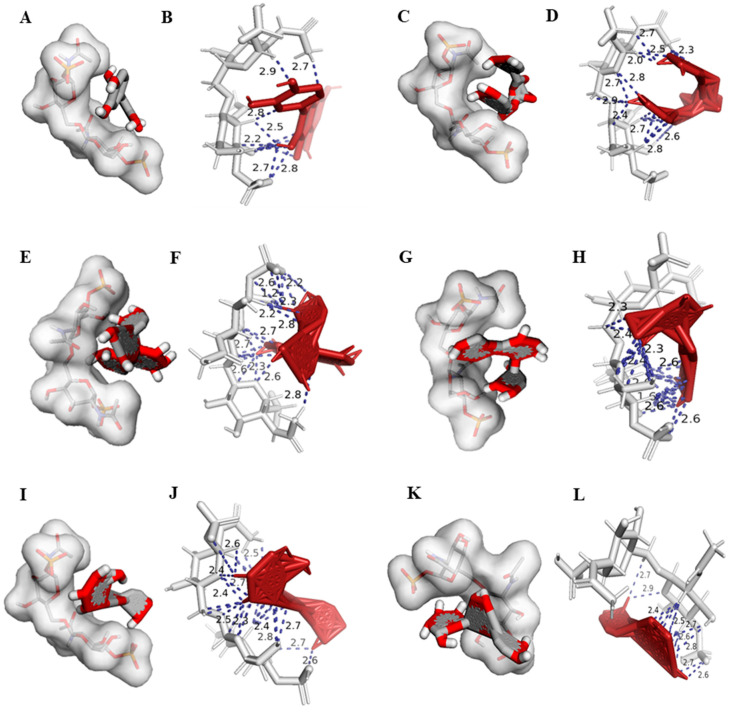
Molecular docking conformations and interaction modes of the compounds with LPS/Lipid A. (**A**,**B**) Quercetin; (**C**,**D**) cichoric acid; (**E**,**F**) rutin; (**G**,**H**) chlorogenic acid; (**I**,**J**) luteolin-7-*O*-glucoside; (**K**,**L**) luteolin. Panels (**A**,**C**,**E**,**G**,**I**,**K**) show the overall docking conformations of each compound within the binding region of LPS/Lipid A, whereas panels (**B**,**D**,**F**,**H**,**J**,**L**) show enlarged views of the local interaction regions.

To further elucidate the molecular basis underlying the potential interactions between the screened phytochemicals and LPS/Lipid A, six representative compounds, namely quercetin, cichoric acid, rutin, chlorogenic acid, luteolin-7-*O*-glucoside, and luteolin, were selected for molecular docking analysis with LPS/Lipid A. As shown in Figure 4 and Table 3, all six compounds were able to form relatively stable docking conformations with LPS/Lipid A, with binding energies ranging from −5.7 to −8.1 kcal/mol. Among them, cichoric acid exhibited the lowest binding energy of −8.1 kcal/mol, suggesting the strongest predicted binding capacity toward LPS/Lipid A. It was followed by chlorogenic acid, quercetin, luteolin-7-*O*-glucoside, rutin, and luteolin, with binding energies of −7.8, −7.1, −6.4, −6.2, and −5.7 kcal/mol, respectively. These results indicate that the screened phenolic acids and flavonoids possess varying degrees of potential binding capacity toward LPS/Lipid A, with cichoric acid and chlorogenic acid showing relatively stronger interaction tendencies.

The observed differences may be attributable to variations in the types, numbers, and spatial distributions of polar functional groups among the compounds. Cichoric acid contains four phenolic hydroxyl groups, two carboxyl groups, and two ester groups, whereas chlorogenic acid contains multiple phenolic and aliphatic hydroxyl groups, together with one carboxyl group and one ester group. These functional groups may form multiple hydrogen bonds and polar contacts with phosphate groups, hydroxyl groups, and glycosidic oxygen atoms in the polar head region of LPS/Lipid A, which may partly account for their relatively favorable predicted binding energies of −8.1 and −7.8 kcal/mol, respectively. Quercetin possesses a relatively planar flavonoid scaffold, five hydroxyl groups, and one carbonyl group, and exhibited a predicted binding energy of −7.1 kcal/mol.

In contrast, although luteolin-7-*O*-glucoside and rutin contain multiple polar groups, glycosylation increases their molecular size and conformational flexibility, which may reduce their effective spatial compatibility with LPS/Lipid A; their predicted binding energies were −6.4 and −6.2 kcal/mol, respectively. Luteolin lacks carboxyl and ester groups and contains comparatively fewer polar functional groups capable of participating in multipoint interactions, resulting in the least favorable predicted binding energy of −5.7 kcal/mol. Collectively, the multipoint interactions supported by the carboxyl, ester, and multiple hydroxyl groups of cichoric acid and chlorogenic acid may constitute an important structural basis for their relatively stronger predicted binding tendencies.

### 3.5. SPR Confirms the Direct Binding of Representative Phytochemicals to LPS

**Figure 5:**
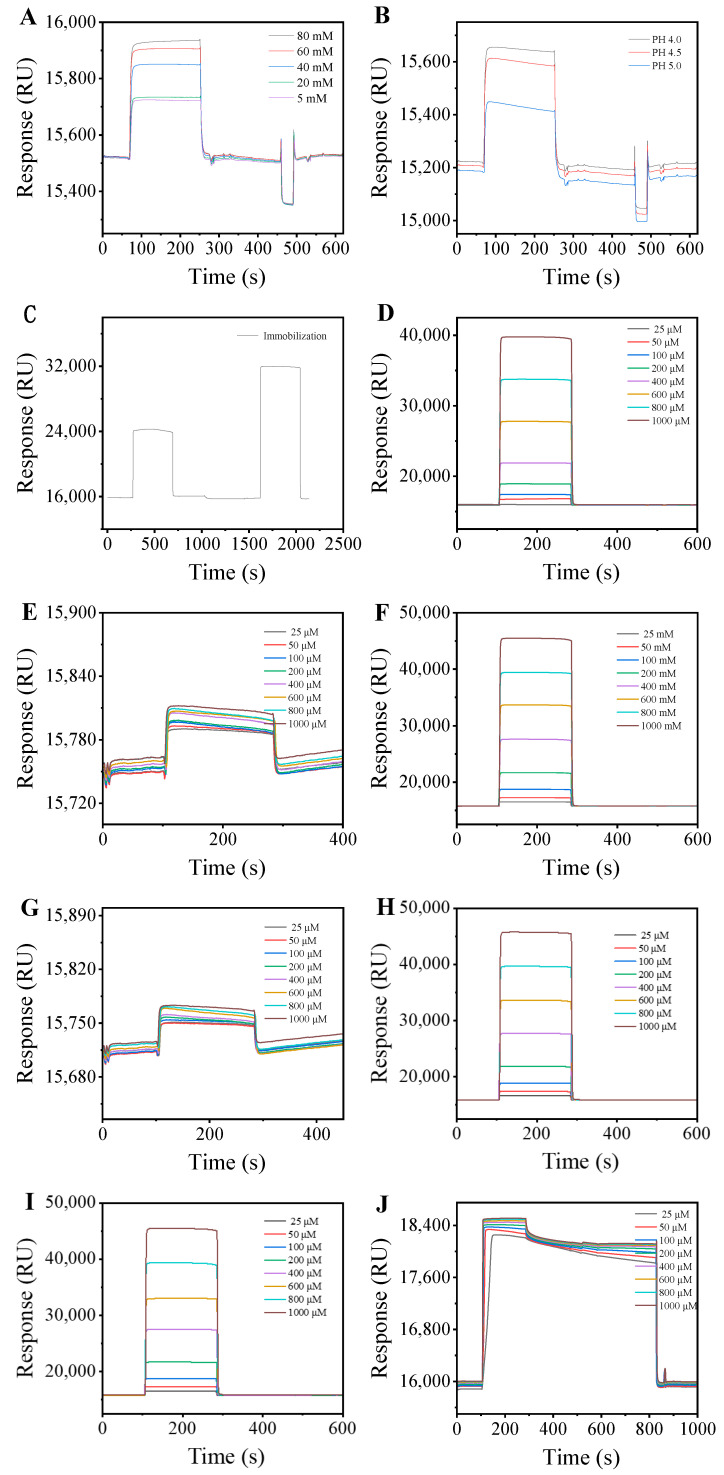
SPR condition optimization and interaction analysis. (**A**) LPS concentration optimization; (**B**) pH optimization; (**C**) LPS immobilization; (**D**) quercetin; (**E**) cichoric acid; (**F**) rutin; (**G**) chlorogenic acid; (**H**) luteolin-7-*O*-glucoside; (**I**) luteolin; (**J**) polymyxin B.

To further verify the direct interactions between representative phytochemicals and LPS, SPR was used to determine their binding kinetic parameters. The LPS immobilization conditions were first optimized. As shown in Figure 5A, different LPS concentrations produced distinct responses on the chip surface. Considering the immobilization response, baseline stability, and requirements for subsequent small-molecule detection, 80 μg/mL was selected as the LPS coupling concentration. The coupling pH was then further optimized at this concentration. As shown in Figure 5B, LPS showed a relatively high and stable response under pH 4.0 conditions; therefore, pH 4.0 was selected as the optimal coupling pH. After immobilization under the conditions of 80 μg/mL and pH 4.0, the chip response increased markedly and remained stable, indicating the successful immobilization of LPS on the sensor chip surface (Figure 5C).

On this basis, the binding behaviors of quercetin, cichoric acid, rutin, chlorogenic acid, luteolin-7-*O*-glucoside, luteolin, and polymyxin B with LPS were further analyzed. As shown in Figure 5D–J and Table 4, all compounds exhibited concentration-dependent SPR responses to varying degrees, although their affinities differed substantially. Polymyxin B, used as the positive control, showed the strongest binding capacity, with a *K*D value of 2.91 × 10^−11^ M, confirming the reliability of this system for evaluating LPS-binding molecules. Among the phytochemicals, chlorogenic acid and cichoric acid exhibited stronger binding capacities. The *k*a, *k*d, and *K*D values of chlorogenic acid were 1.21 × 10^6^ M^−1^·s^−1^, 0.008942 s^−1^, and 7.42 × 10^−9^ M, respectively. Cichoric acid showed a higher *k*a value than chlorogenic acid, at 2.37 × 10^6^ M^−1^·s^−1^, but also had a higher *k*d value of 0.06236 s^−1^, resulting in a *K*D value of 2.63 × 10^−8^ M. These results suggest that cichoric acid binds more rapidly, whereas chlorogenic acid forms a more stable complex with LPS.

The other flavonoid compounds showed relatively weaker binding capacities. The *K*D values of quercetin, luteolin-7-*O*-glucoside, luteolin, and rutin were 5.53 × 10^−4^, 0.007495, 0.03005, and 0.4918 M, respectively. The overall affinity order was as follows: polymyxin B >> chlorogenic acid >> cichoric acid >> quercetin > luteolin-7-*O*-glucoside > luteolin > rutin. These results indicate that phenolic acids possess stronger LPS-binding potential than flavonoids, which may be related to the ability of their carboxyl, ester, and phenolic hydroxyl groups to form hydrogen bonds and polar interactions with phosphate groups, hydroxyl groups, or glycosidic oxygen atoms in LPS.

### 3.6. Representative LPS-Binding Compounds Exhibit Antibacterial Activity Against Gram-Negative Bacteria

**Figure 6:**
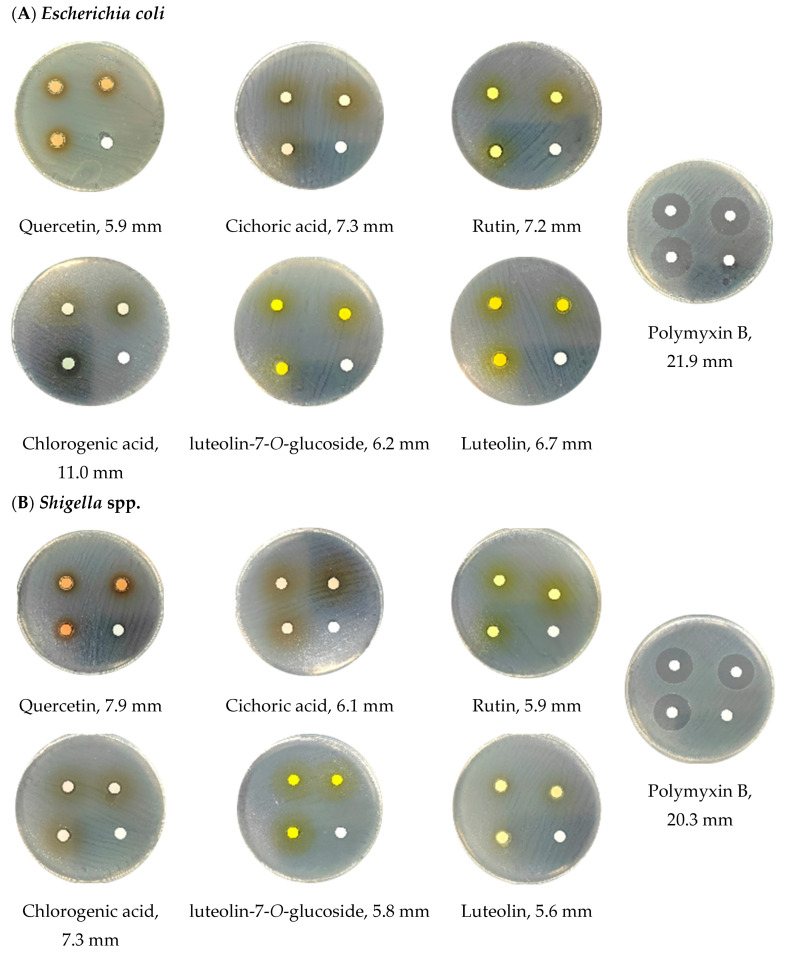
Inhibition zone assay results of different compounds against *Escherichia coli* and *Shigella* spp. (**A**) *Escherichia coli*; (**B**) *Shigella* spp.

To further evaluate the growth-inhibitory effects of representative LPS/Lipid A-binding compounds against Gram-negative bacteria, the antibacterial activities of each compound against *Escherichia coli* and *Shigella* spp. were examined using the inhibition zone assay and broth microdilution method. As shown in Figure 6, polymyxin B, used as the positive control, exhibited pronounced antibacterial activity against both Gram-negative bacterial strains, with inhibition zone diameters of 21.9 mm and 20.3 mm against *E. coli* and *Shigella* spp., respectively, indicating good responsiveness and reliability of the experimental system.

In the *E. coli* model, the plant-derived small molecules showed varying degrees of antibacterial activity. Among them, chlorogenic acid produced the largest inhibition zone, with a diameter of 11.0 mm, suggesting a relatively more pronounced inhibitory effect on *E. coli* growth. The inhibition zone diameters of cichoric acid, rutin, luteolin, luteolin-7-*O*-glucoside, and quercetin were 7.3, 7.2, 6.7, 6.2, and 5.9 mm, respectively, indicating that these compounds also exhibited certain inhibitory tendencies against *E. coli*, although their overall effects were weaker than that of the positive control. Combined with the MIC results, the MIC of cichoric acid against *E. coli* was 1024 μg/mL, indicating that it could inhibit *E. coli* growth at a relatively high concentration.

In the *Shigella* spp. model, the overall antibacterial activity of the plant-derived small molecules was weaker, but certain differences were still observed. Quercetin showed the largest inhibition zone, with a diameter of 7.9 mm, followed by chlorogenic acid, with an inhibition zone diameter of 7.3 mm. The inhibition zone diameters of cichoric acid, rutin, luteolin-7-*O*-glucoside, and luteolin were 6.1, 5.9, 5.8, and 5.6 mm, respectively (Table 5). Although cichoric acid did not produce the largest inhibition zone in the plate diffusion assay against *Shigella* spp., MIC determination further showed that its MIC against *Shigella* spp. was also 1024 μg/mL (Table 6). These results indicate that cichoric acid had an MIC value of 1024 μg/mL against both tested strains, suggesting detectable growth-inhibitory activity at relatively high concentrations.

Overall, the representative LPS/Lipid A-binding compounds exhibited certain antibacterial activities against both *E. coli* and *Shigella* spp., but their direct antibacterial effects were markedly weaker than that of polymyxin B. Notably, cichoric acid showed an MIC value of 1024 μg/mL against both *E. coli* and *Shigella* spp., indicating a relatively consistent antibacterial tendency. Combined with the preceding SPR results, cichoric acid not only directly interacted with LPS/Lipid A but also exhibited detectable inhibitory activity in bacterial growth assays, suggesting that it may exert biological activity by interfering with Gram-negative bacterial outer membrane-related structures or LPS/Lipid A-mediated processes.

## 4. Discussion

### 4.1. Significance of the LPS-Targeted Affinity Platform for Screening Active Constituents from Taraxacum mongolicum

LPS is an essential structural component of the outer membrane of Gram-negative bacteria and a key pathogenic factor responsible for endotoxin-induced inflammatory responses [1,3,4,5]. Small-molecule compounds capable of directly recognizing or binding LPS/Lipid A may have potential value in disturbing bacterial outer membrane homeostasis and attenuating LPS-associated inflammatory responses. Polymyxin antibiotics are classical LPS-targeting molecules, and their antibacterial effects are closely associated with their binding to LPS/Lipid A and subsequent disruption of the outer membrane structure [16,17]. This provides a clear pharmacological rationale for screening natural active constituents using LPS as a target.

In this study, an LPS-immobilized Ag@SiO_2_ plasmonic core–shell affinity screening platform was constructed to selectively enrich potential LPS-binding constituents from the complex extract of *T. mongolicum*. Unlike conventional bioactivity-guided isolation strategies that rely primarily on endpoint pharmacological effects, this approach uses LPS as a defined molecular target and prioritizes compounds with direct binding potential through affinity capture. This strategy is consistent with the emerging concepts of affinity selection-mass spectrometry and bioaffinity screening in natural product-based drug discovery. Raman spectroscopy provides rapid and nondestructive molecular fingerprinting and has been widely applied to chemical characterization and analytical process monitoring [18]. TEM, EDS, and Raman/SERS results demonstrated that Ag NPs were successfully converted into the Ag@SiO_2_-NH_2_-LPS nanoplatform after SiO_2_ coating, amino functionalization, and LPS conjugation. In contrast to previous SERS studies that mainly focused on LPS or endotoxin detection [13,14], the present study further applied the SERS/Raman platform to LPS immobilization, affinity enrichment, and ligand–interface interaction analysis, thereby expanding the application scope of SERS/Raman-based platforms in targeted natural product screening.

### 4.2. Chemical Basis and Potential Binding Mechanisms of LPS-Binding Constituents from Taraxacum mongolicum

Some of the candidate constituents identified in the present study are consistent with previous phytochemical investigations of *Taraxacum mongolicum*. Using HPLC, Duan et al. analyzed different medicinal parts of *T. mongolicum* and demonstrated that both chlorogenic acid and cichoric acid were detectable in the flowers, leaves, and roots, with cichoric acid being one of the relatively abundant phenolic acids [19]. Li et al. further evaluated 15 batches of *T. mongolicum* samples using HPLC fingerprinting combined with multi-component quantitative analysis, and likewise identified and quantified chlorogenic acid and cichoric acid [20]. In addition, a recent study of *T. mongolicum* leaves collected from different geographical regions included chlorogenic acid, caffeic acid, and cichoric acid among the major phenolic acids analyzed [21]. Therefore, the detection of chlorogenic acid and cichoric acid in the *T. mongolicum* extract in the present study is supported by a well-established phytochemical basis. In contrast to previous studies that primarily focused on constituent identification and quantitative determination, the present study further employed an LPS-immobilized affinity platform to selectively enrich these known constituents and evaluated their potential interactions with LPS using SPR and complementary approaches.

Based on affinity enrichment using the LPS-immobilized platform combined with UPLC-MS analysis, multiple potential LPS-binding constituents were identified from *T. mongolicum* extract, mainly including phenolic acids, flavonoids, coumarins, and fatty acids. Among them, phenolic acids and flavonoids accounted for the major proportion, suggesting that polyphenolic compounds may represent an important material basis underlying the LPS-targeted activity of *T. mongolicum*. Previous studies have shown that plants of the genus Taraxacum are rich in bioactive constituents, including polyphenols, flavonoids, coumarins, and terpenoids, which are associated with pharmacological effects such as anti-inflammatory, antioxidant, antibacterial, and immunomodulatory activities. By screening and validating these constituents from the perspective of LPS binding, the present study provides a more specific material basis for explaining the anti-infective and potential anti-endotoxin activities of *T. mongolicum*.

From a structural perspective, chlorogenic acid, cichoric acid, rutin, quercetin, luteolin-7-*O*-glucoside, and luteolin all contain structural moieties such as phenolic hydroxyl, carboxyl, ester, glycosyl, carbonyl, or aromatic skeletons. These groups may participate in the recognition of LPS/Lipid A through hydrogen bonding, polar interactions, electrostatic-related interactions, and spatial complementarity. The polar head region of Lipid A contains phosphate groups and a glucosamine backbone, which may provide potential hydrogen-bonding and polar interaction sites for polyphenolic compounds, whereas the fatty acyl chain region may contribute to hydrophobic or van der Waals interactions.

The molecular docking results further support this inference. All six representative compounds formed relatively stable docking conformations with LPS/Lipid A, with binding energies ranging from −5.7 to −8.1 kcal/mol. Among them, cichoric acid exhibited the lowest binding energy of −8.1 kcal/mol, indicating the strongest theoretical binding potential, followed by chlorogenic acid, quercetin, luteolin-7-*O*-glucoside, rutin, and luteolin, with binding energies of −7.8, −7.1, −6.4, −6.2, and −5.7 kcal/mol, respectively. The abundant phenolic hydroxyl, carboxyl, and ester groups in cichoric acid and chlorogenic acid may enable multipoint interactions with the polar head region of LPS/Lipid A, which may partly explain their lower binding energies. In contrast, although rutin and luteolin-7-*O*-glucoside contain multiple polar groups, their larger molecular sizes and higher conformational flexibility may weaken spatial compatibility. This suggests that LPS-binding capacity is determined not only by the number of polar functional groups but also by molecular geometry and the effective contact area.

### 4.3. Relationship Between LPS-Binding Capacity, Anti-Gram-Negative Bacterial Activity, and Potential Anti-Endotoxin Effects

SPR enables real-time and label-free monitoring of molecular interactions and has been widely applied to affinity and kinetic analyses of biomolecular recognition events [22]. In the present study, SPR further supported the interactions between the representative compounds and immobilized LPS. Polymyxin B, used as the positive control, showed the strongest apparent binding capacity, which is consistent with its known mechanism of recognizing LPS/Lipid A and disrupting the outer membrane structure of Gram-negative bacteria [15,16]. This also confirms that the SPR system established in this study is suitable for evaluating the binding behavior between candidate small molecules and LPS. Among the plant-derived compounds, chlorogenic acid and cichoric acid exhibited relatively strong apparent affinities, with fitted KD values of 7.42 × 10^−9^ M and 2.63 × 10^−8^ M, respectively, suggesting that they may represent typical LPS-binding candidate compounds in *T. mongolicum*. Previous studies have shown that chlorogenic acid can attenuate inflammatory and oxidative stress responses in LPS-induced RAW264.7 macrophage inflammatory models, and cichoric acid has also been reported to alleviate LPS-induced inflammatory injury by regulating the TLR4/MAPK/NF-κB pathway [23,24]. These literature findings are generally consistent with the LPS-binding tendency observed in the present study, suggesting that phenolic acids may contribute substantially to the LPS-targeted activity of *T. mongolicum*. In contrast, quercetin, luteolin-7-*O*-glucoside, luteolin, and rutin showed relatively weaker apparent SPR affinities, indicating that the LPS-binding capacity of different polyphenolic constituents may be jointly influenced by molecular configuration, the number of hydroxyl/carboxyl groups, glycosylation modification, and spatial compatibility. It should be noted that immobilized LPS is structurally heterogeneous, and SPR results may be affected by immobilization density, mass transfer, compound solubility, refractive index variation, and nonspecific adsorption. Therefore, the kinetic parameters obtained by fitting with the 1:1 Langmuir model should be interpreted primarily as apparent binding parameters rather than absolute affinity constants in a homogeneous system. Molecular docking and SPR provide theoretical binding conformations and direct experimental binding evidence, respectively, and should be considered complementary evidence supporting the possibility that the candidate compounds target LPS.

The direct antibacterial activities of the candidate compounds examined in this study were lower than expected, as their MIC values were generally high. Chlorogenic acid and cichoric acid both exhibited MIC values of 1024 μg/mL against *Escherichia coli*, whereas the MIC values of cichoric acid and quercetin against *Shigella* spp. were 1024 and 2048 μg/mL, respectively, indicating that these compounds produced detectable growth inhibition only at relatively high concentrations. Lou et al. reported MIC values of 80 and 20 μg/mL for chlorogenic acid against *E. coli* and *Shigella dysenteriae*, respectively [25]. Jaisinghani and Mansukhani reported that quercetin inhibited *E. coli* at 400 μg/mL, whereas no detectable inhibition of *Shigella flexneri* was observed at concentrations up to 500 μg/mL [26]**.** These discrepancies may be attributable to differences in bacterial species and strains, inoculum sizes, culture conditions, solvent systems, and experimental methodologies. Taken together, the SPR and antibacterial results showed that chlorogenic acid and cichoric acid exhibited relatively strong apparent affinities for LPS and comparatively pronounced antibacterial effects under the present experimental conditions, suggesting that phenolic acids may possess stronger LPS-binding tendencies and greater anti-Gram-negative bacterial potential than flavonoids. However, this trend is based on a limited number of compounds, and the current evidence does not establish LPS binding as the direct mechanism underlying their antibacterial effects. Further experimental validation is therefore required.

To further contextualize our findings, we compared our results with previous studies on the antibacterial and LPS-related activities of chlorogenic acid, cichoric acid, quercetin, and related phenolic compounds. Previous studies have demonstrated that chlorogenic acid exerts anti-Gram-negative activity by disrupting bacterial membrane integrity, inducing membrane depolarization and ATP depletion, and its antibacterial effect can be synergistically enhanced when combined with lysozyme [27,28]. In addition, chlorogenic acid has been reported as a quorum sensing inhibitor that suppresses bacterial biofilm formation and virulence expression by competitively binding to AHL receptors or targeting the LuxS enzyme in the AI-2 signaling pathway [29]. In LPS-induced inflammatory models, chlorogenic acid isomers (3-CQA, 4-CQA, 5-CQA) significantly reduced the levels of pro-inflammatory mediators such as TNF-α, IL-6, NO, and ROS through mechanisms involving stable binding to and inhibition of AKR1B1, as well as enhancement of cholinergic anti-inflammatory signaling via acetylcholinesterase inhibition and muscarinic receptor activation [30]. The multi-target anti-inflammatory and antibacterial properties of chlorogenic acid and its derivatives have been systematically reviewed [31]. Cichoric acid has been shown to alleviate LPS-induced inflammatory injury by regulating the TLR4/NF-κB pathway and to inhibit LPS-induced ferroptosis via the Nrf2/HO-1 signaling axis [32]. Molecular docking studies of major phenolic constituents from *Taraxacum* species, including chlorogenic acid and cichoric acid, with MD-2 protein have also suggested that these compounds may exert anti-inflammatory effects by competing with LPS for MD-2 binding [32]. Quercetin has been demonstrated to directly bind to virulence proteins of various Gram-negative bacteria, inhibit virulence factor expression and biofilm formation, and act as an efficient inhibitor of *Pseudomonas aeruginosa* LasB elastase, while also significantly reducing biofilm formation and swimming motility through quorum sensing inhibition [33,34]. In the present study, all three compounds exhibited direct affinity for LPS, with the order of binding strength being chlorogenic acid > cichoric acid > quercetin (based on SPR KD values of 7.42 × 10^−9^ M, 2.63 × 10^−8^ M, and 6.44 × 10^−7^ M, respectively), which is generally consistent with the reported mechanisms by which they exert anti-inflammatory effects through targeting MD-2 protein or the TLR4 pathway. However, our findings also suggest that LPS itself may be a direct target of these phenolic compounds, extending the current understanding of their mechanisms of action. Moreover, in contrast to studies focusing on single phenolic compounds, the multi-component synergistic effects present in *T. mongolicum* may further enhance its anti-Gram-negative potential, an aspect that has received less attention in previous reports.

### 4.4. Limitations and Future Perspectives

Although this study established an integrated evidence chain combining LPS-targeted affinity screening, UPLC-MS-based constituent identification, molecular docking, SPR validation, and antibacterial evaluation, several limitations remain. First, LPS is a highly complex biomacromolecule with pronounced source-dependent structural heterogeneity. LPS derived from different bacterial strains may vary in Lipid A acylation, phosphate modification, core oligosaccharide composition, and O-antigen structure. Although the immobilized LPS model can reflect direct binding behavior, it cannot fully recapitulate the native arrangement of LPS and the membrane microenvironment in the bacterial outer membrane [2,5]. Second, the LPS immobilization process may alter the exposure of certain binding sites, and some SPR fitting results showed relatively low (Rmax) values or high U-values. Therefore, the derived (KD), (ka), and (kd) values should be interpreted as apparent kinetic parameters rather than absolute thermodynamic constants. Third, the present study was primarily based on in vitro affinity screening, SPR binding analysis, and preliminary antibacterial evaluation, without assessments of mammalian cytotoxicity, hemolytic activity, or in vivo efficacy. Therefore, the cytocompatibility, selectivity index, safety margin, bioavailability, and in vivo effectiveness of the candidate compounds cannot currently be determined. Given the relatively high MIC values of some candidate compounds, it also remains unclear whether acceptable safety can be maintained at concentrations required to achieve antibacterial effects. Fourth, the current antibacterial assays only demonstrated that some representative compounds exerted detectable growth-inhibitory effects at relatively high concentrations. However, it remains uncertain whether these effects are associated with disruption of the bacterial outer membrane, alteration of LPS homeostasis, or other mechanisms. Accordingly, the available evidence is insufficient to establish a direct mechanistic link between LPS binding and antibacterial activity.

## 5. Conclusions

This study established an LPS-immobilized Ag@SiO_2_ affinity screening platform and, in combination with UPLC-Q-Orbitrap HRMS/MS, annotated 14 potential LPS-binding constituents from *Taraxacum mongolicum* extract, mainly phenolic acids and flavonoids. The six representative constituents exhibited predicted binding energies ranging from −5.7 to −8.1 kcal/mol, with cichoric acid and chlorogenic acid showing relatively favorable predicted binding tendencies at −8.1 and −7.8 kcal/mol, respectively. SPR analysis further confirmed the direct interactions of the representative compounds with immobilized LPS. Among them, chlorogenic acid and cichoric acid exhibited relatively strong apparent affinities, with *K*D values of 7.42 × 10^−9^ and 2.63 × 10^−8^ M, respectively. Antibacterial assays showed that the direct antibacterial activities of these candidate compounds were generally limited, with cichoric acid exhibiting an MIC of 1024 μg/mL against both *Escherichia coli* and *Shigella* spp. Taken together, chlorogenic acid and cichoric acid may be regarded as representative LPS-binding candidates in *T. mongolicum*; however, the available evidence remains insufficient to establish LPS binding as the direct mechanism underlying their antibacterial effects. This study establishes an integrated workflow comprising target-directed affinity screening, constituent identification, binding validation, and preliminary functional evaluation, thereby providing a methodological basis for the discovery of LPS-binding natural small molecules from complex plant extracts.

## Figures and Tables

**Table 1:** Quantitative elemental composition of the samples at different modification stages determined by EDS.

Sample	Ag (wt%)	Si (wt%)	O (wt%)	C (wt%)	N (wt%)
Ag NPs	44.15	ND	9.64	46.21	ND
Ag@SiO_2_	20.33	25.04	23.26	31.37	ND
Ag@SiO_2_-NH_2_	18.36	23.48	21.72	34.18	2.26
LPS-Ag@SiO_2_	8.50	14.37	26.91	46.71	3.51

**Table 2:** Screening results of lipopolysaccharide-targeted active constituents from *Taraxacum mongolicum*.

No.	Compound	Molecular Formula	Molecular Weight	Observed Ion (*m*/*z*)	Ion Type	Characteristic Fragment Ion (*m*/*z*)
1	Caffeic acid	C_9_H_8_O_4_	180.16	179.6	[M − H]^−^	134.71
2	Chlorogenic acid	C_16_H_18_O_9_	354.31	353.4	[M − H]^−^	190.75
3	Monocaffeoyltartaric acid	C_13_H_12_O_9_	312.23	312.2	[M − H]^−^	135.91
4	Luteolin	C_15_H_10_O_6_	286.24	285.2	[M − H]^−^	217.03
5	Quercetin	C_15_H_10_O_7_	302.24	301	[M − H]^−^	152.04
6	Rutin	C_27_H_30_O_16_	610.52	609.1	[M − H]^−^	301.24
7	Ferulic acid	C_10_H_10_O_4_	194.18	193.1	[M − H]^−^	177.25
8	p-Coumaric acid	C_9_H_8_O_3_	164.16	163.1	[M − H]^−^	119.61
9	Cichoric acid	C_22_H_18_O_12_	474.37	473.38	[M − H]^−^	311.09, 191.14
10	Luteolin-7-*O*-glucoside	C_21_H_20_O_11_	448.38	447.2	[M − H]^−^	284.13
11	Hyperoside	C_21_H_20_O_12_	464.37	463.2	[M − H]^−^	300.21
12	Isoquercitrin	C_21_H_20_O_12_	464.48	463.9	[M − H]^−^	299.76
13	Esculetin	C_9_H_6_O_4_	178.14	176.8	[M − H]^−^	132.92
14	Behenic acid	C_22_H_44_O_2_	340.58	339.58	[M − H]^−^	323.52

**Table 3:** Molecular docking results of representative compounds with LPS/Lipid A.

No.	Compound	Molecular Formula	Formula Molecular Weight	Weight Binding Energy (kcal/mol)
1	Quercetin	C_15_H_10_O_7_	302.24	−7.1
2	Cichoric acid	C_22_H_18_O_12_	474.37	−8.1
3	Rutin	C_27_H_30_O_16_	610.52	−6.2
4	Chlorogenic acid	C_16_H_18_O_9_	354.309	−7.8
5	Luteolin-7-*O*-glucoside	C_21_H_20_O_11_	448.38	−6.4
6	Luteolin	C_15_H_10_O_6_	286.24	−5.7

**Table 4:** SPR-derived kinetic and affinity parameters of different compounds binding to immobilized LPS.

Compound	k_a_ (M^−1^·s^−1^)	k_d_ s^−1^ (s^−1^)	K_D_ (M)	Rmax (RU)	Chi^2^ (RU^2^)	U-Value
Quercetin	1298	0.718	5.53 × 10^−4^	1.58 × 10^4^	1.65 × 10^5^	15
Cichoric acid	2.37 × 10^6^	0.06236	2.63 × 10^−8^	0.007579	16.3	95
Rutin	2.366	1.164	0.4918	1.23 × 10^7^	1.59 × 10^4^	3
Chlorogenic acid	1.21 × 10^6^	0.008942	7.42 × 10^−9^	0.004165	20.9	95
Luteolin-7-*O*-glucoside	156.7	1.175	0.007495	1.98 × 10^5^	8.10 × 10^3^	9
Luteolin	42.6	1.28	0.03005	3.65 × 10^5^	1.26 × 10^4^	11
Polymyxin B	9.50 × 10^9^	0.2765	2.91 × 10^−11^	2352	1.89 × 10^3^	95

**Table 5:** Inhibition zone diameters of different compounds against *Escherichia coli* and *Shigella* spp.

No.	Compound	Inhibition Zone Diameter Against *E. coli* (mm)	Inhibition Zone Diameter Against *Shigella* spp. (mm)
1	Chlorogenic acid	11.0	7.3
2	Cichoric acid	7.3	6.1
3	Rutin	7.2	5.9
4	Quercetin	5.9	7.9
5	Luteolin-7-*O*-glucoside	6.2	5.8
6	Luteolin	6.7	5.6

**Table 6:** MIC values of the screened compounds.

Species	Compound	Concentration (μg/mL)												
		4096	2048	1024	512	256	128	64	32	16	8	4	2	MIC
*Shigella* spp.	Cichoric acid	+	+	+	−	−	−	−	−	−	−	−	−	1024
	Quercetin	+	+	−	−	−	−	−	−	−	−	−	−	2048
	Polymyxin B	+	+	+	+	+	+	+	+	+	+	+	+	≤2
*Escherichia coli*	Cichoric acid	+	+	+	−	−	−	−	−	−	−	−	−	1024
	Chlorogenic acid	+	+	+	−	−	−	−	−	−	−	−	−	1024
	Polymyxin B	+	+	+	+	+	+	+	+	+	+	+	+	≤2

Note: “+” indicates a positive result, and “−” indicates a negative result.

## Data Availability

The main data supporting the findings of this study are included in the article. Additional relevant data are available from the corresponding author upon reasonable request.
